# The Importance of Humic Acids in Shaping the Resistance of Soil Microorganisms and the Tolerance of *Zea mays* to Excess Cadmium in Soil

**DOI:** 10.3390/ijms262412175

**Published:** 2025-12-18

**Authors:** Agata Borowik, Jadwiga Wyszkowska, Magdalena Zaborowska, Jan Kucharski

**Affiliations:** Department of Soil Science and Microbiology, Faculty of Agriculture and Forestry, University of Warmia and Mazury in Olsztyn, 10-719 Olsztyn, Poland; agata.borowik@uwm.edu.pl (A.B.); m.zaborowska@uwm.edu.pl (M.Z.); jan.kucharski@uwm.edu.pl (J.K.)

**Keywords:** rhizosphere, soil bacteriobiome, functional predictions, *maize*, cadmium, Humus Active, 16S rRNA, bioremediation

## Abstract

Contamination with cadmium (Cd^2+^) poses a severe threat to the soil environment due to its toxic effect on bacteria, being of key importance to soil fertility and plant health. The present study aimed to evaluate the effect of a humic preparation, Humus Active (HA), on the structure, diversity, and functional potential of soil bacteria under conditions of cadmium stress during *Zea mays* cultivation. A model study was conducted to analyze the response of bacteria to soil contamination with 60 mg Cd kg^−1^ under conditions of soil fertilization with humic acid at doses of 2 g (HA2) and 4 g (HA4) kg^−1^ of soil. Microbiological analyses were carried out with both culture and non-culture (16S rRNA gene amplicon sequencing method) methods. Bacteria function prediction was also performed using FAPROTAX software. The study results demonstrated that Cd caused a 92% reduction in *Zea mays* biomass and a significant decrease (by 52%) in the abundance of organotrophic bacteria. The NGS analysis showed that it also reduced the population of the *Neobacillus* bacteria in the soil (by 50%), simultaneously causing an over twofold increase in the population of the *Nocardioides* genus bacteria. The application of HA (particularly in the HA4 dose) substantially mitigated Cd phytotoxicity. In the Cd-contaminated soil, HA4 stimulated the growth of culturable actinobacteria. The soil bacteria community was predominated by chemoheterotrophic bacteria and the nitrogen cycle bacteria, driven by tolerant, Cd^2+^-resistant bacteria from the following genera: *Bacillus*, *Nocardioides*, and *Arthrobacter*. The study results enable concluding that even though Humus Active does not restore the original microbiome structure, it promotes the development of a new stress-resistant bacterial community exhibiting high bioremediating potential, thereby directly translating into improved plant condition. Subsequently, humic acids provide an innovative approach that not only extends knowledge about the mechanisms behind bacterial resistance but also enables developing practical methods for diminishing cadmium mobility in the soil.

## 1. Introduction

Cadmium is one of the toxic metals most frequently detected in agricultural and urbanized soils [1,2]. The United Nations Food and Agriculture Organization (FAO) warns that up to 90% of the world’s soil resources may be at risk due to excessive degradation by 2050 [3,4]. Its presence in soils has both geological and anthropogenic origins, including the use of certain phosphate fertilizers and industrial sludge [5,6]. In particular, phosphate raw materials and fertilizers produced from certain phosphorite deposits are the major sources of its deposition in agricultural soils [5]. Its mobility and bioavailability depend on pH, soil organic matter content, the sorptive properties of clay minerals, as well as the presence of competing anions and cations [7]. For this reason, managing the bioavailable fraction of Cd is crucial for diminishing its transfer into the food chain [8,9]. The mobilization and immobilization of Cd are processes of considerable practical importance from the perspective of soil contamination management [10,11,12,13], as the first may increase its uptake by plants and leaching into deeper soil layers, while the latter minimizes its transfer into the food chain and reduces its leaching [13,14]. Despite its widespread occurrence in the environment, most cadmium in soils is present in poorly available forms because its substantial fraction occurs as sparingly soluble mineral compounds [15,16].

Environmental pressure leads to changes in the structure of the microbial community—sensitive species are successively replaced by a few resistant forms, which may disrupt soil biochemical processes and reduce the bioavailability of elements essential for plant growth [17,18,19]. Thus, cadmium has an immediate effect on the quality and safety of agricultural production [20]. It can be readily taken up by plant root systems and accumulated in their tissues, often at concentrations exceeding permissible levels. [21,22]. Acceptable concentrations of Cd in agricultural soils vary depending on crop type and health risk assessment [20]. For rice and wheat, permissible values range from 0.01 to 0.4 mg kg^−1^, whereas for cocoa grains, they may reach up to 0.8 mg kg^−1^ [23,24].

Cadmium-tolerant soil bacteria exhibit significant phylogenetic and functional diversity [23,25,26]. They have recently become more important because of their vital ecological functions, and their ability to persist in Cd-enriched environments is ecologically significant, even though they do not form a homogeneous taxonomic group. According to Bravo and Braissant [23] and Xia et al. [13], bacteria showing the highest resistance to Cd belong mainly to Proteobacteria and Firmicutes phyla, which suggests their substantial involvement in bacteria adaptation to the environment exposed to heavy metals [20,27,28,29], and the most Cd-tolerant of them include those belonging to *Pseudarthrobacter*, *Desulfurivibrio*, *Thiobacillus*, and *Sulfurimonas* [30] as well as *Burkholderia*, *Herbaspirillum*, *Pantoea*, *Pseudomonas*, and *Paenibacillus* genera [12,28]. As Guo et al. [31] and Luo et al. [32] claim, their high tolerance of this heavy metal is due to mechanisms based on biosorption and bioaccumulation. In the scientific literature, they are referred to as “resistant”, “tolerant” [33] or “immobilizing” [29]. Their tolerance mechanisms include, among other things, biofilm formation, biosorption of metal ions, active Cd pumping out of a cell, and bioconversion of Cd to less toxic forms [12,32,33]. One of the important adaptive strategies of bacteria is also the expression of metal-transporting genes. Among the most commonly identified genes associated with Cd resistance are *czcA*, *czcD*, *zntA*, *cadA*, and *cadD*, which encode efflux pumps or ATPases that transport metals out of the cell [34].

Natural organic substances, particularly humic, huminic, and fulvic acids, have recently spurred increased attention as agents capable of reducing the bioavailability of heavy metals in soils [35,36,37]. Humic substances exhibit a high capacity for forming complexes with metal ions due to the presence of numerous reactive functional groups, including carboxyl and phenolic moieties [37]. Their application can decrease the bioavailable fraction of Cd, reduce its accumulation in edible plant tissues, and improve plant growth and yield under metal-induced stress conditions.

However, research findings regarding the effectiveness of humic acids remain somewhat inconsistent. Studies by Coles and Young [38] and Evangeolu et al. [39] indicate that excessive doses or inadequate chemical composition of humic preparations induce a metal immobilization effect that depends largely on the quality of the humus, application rate, soil conditions, and the species of crop grown. Concentrated humic substances can improve soil structure and the activity of its microbiome, which has bioremediation potential, as well as increasing the availability of nutrients for plants. Ma et al. [40] and Song et al. [41] argue that the use of such preparations is justified as part of an integrated strategy to reduce the bioavailability of heavy metals in soils.

In light of these considerations, the present study aimed to evaluate the effect of a Humus Active preparation on the bacteria (bacteriobiome) constituting part of the microbiome, colonizing soil exhibiting an increased content of cadmium, under conditions of *Zea mays* cultivation. The main objectives focus on determining the taxonomic structure and diversity of bacterial communities in soils with different levels of cadmium contamination based on 16S amplicon sequencing analyses and bacterial cultures on solid media (1), and assessing the metabolic and functional potential of the soil microbiome, focusing on pathways related to metal stress and response to Cd (2). The results of the study will also provide a basis for identifying potential bacterial strains of application importance in biotechnology and environmental protection and for evaluating the effect of Humus Active on the functional restoration of the Cd-contaminated soil microbiome. The innovative aspect of the research is to provide data on the interaction of Cd with Humus Active in the context of the microbiome, and most importantly, to identify potential strains with high bioremediation potential, which is lacking in the scientific international arena. Furthermore, improved recognition of the effectiveness of humic substances in reducing the bioavailability of Cd is crucial for agriculture, since even small changes in the mobility of this element determine food safety. In practice, this provides a real opportunity to improve soil management.

## 2. Results

### 2.1. Effect of Soil Contamination with Cadmium (Cd) on the Abundance and Diversity of Culturable Bacteria

**Figure 1:**
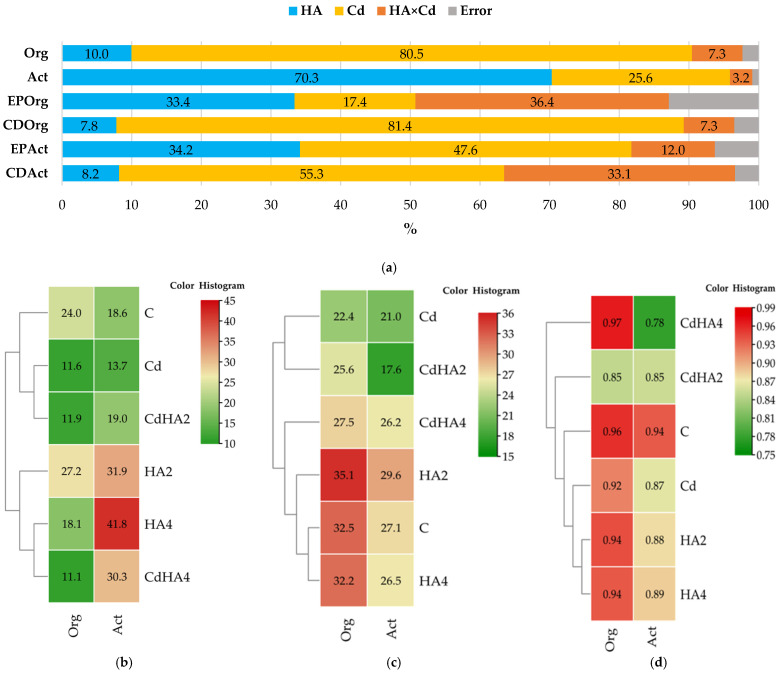
The analysis (**a**) of the effect size (η^2^), (**b**) the abundance of organotrophic bacteria (Org) and actinobacteria (Act) in the soil (10^9^ cfu); (**c**) their colony development (CD) indices; and (**d**) their ecophysiological diversity (EP) indices. Org: organotrophic bacteria; Act: actinobacteria; C: non-contaminated soil; Cd: soil contaminated with cadmium; HA2—Humus Active dose of 2 g per kg of soil; HA4—Humus Active dose of 4 g per kg of soil; CdHA2—soil contaminated with cadmium and amended with a Humus Active dose of 2 g per kg of soil; and CdHA4—soil contaminated with cadmium and amended with a Humus Active dose of 4 g per kg of soil.

The present study demonstrated diversified abundance of organotrophic bacteria and actinobacteria, depending on soil contamination with cadmium and application of the Humus Active preparation (Figure 1a). The effect size (η^2^) analysis revealed a differentiated contribution of the experimental factors to the variance of the examined parameters [42]. In the case of the organotrophic bacteria (Org) abundance, this contribution was determined to the greatest extent by soil contamination with cadmium (Cd) (80.5%). The application of Humus Active (HA) explained barely 10.0% of the total variance, whereas HA × Cd interaction had a marginal share in this respect, explaining only 7.3% of the total variance. In the case of actinobacteria (Act), the highest η^2^ value was noted for HA (70.3%), pointing to a strong stimulating effect of this preparation on the development of this bacterial group. Soil contamination with cadmium was responsible for 25.6% of the variability in actinobacteria abundance, whereas the effect of HA × Cd interaction was negligible (3.2%). The colony development (CD) index was mainly determined by soil contamination with cadmium (in 81.4% for Org and 55.3% for actinobacteria), whereas changes in bacteria EP were triggered by both Cd effect and HA treatment. In the case of Org, the coefficient of determination reached 36.4% for Cd and 17.4% for HA, whereas respective values determined for Act were 47.6% and 34.2%.

The abundance of organotrophic bacteria in the non-contaminated soil (C) reached 24.02 × 10^9^ cfu kg^−1^ d.m. of soil. (Figure 1b), whereas that of actinobacteria—18.64 × 10^9^ cfu kg^−1^. The introduction of cadmium to soil reduced counts of both groups of bacteria by 51.8% and 26.6%, respectively, compared to the control treatment. Non-contaminated soil amendment with the lower tested dose of Humus Active preparation (HA2) contributed to a 13.3% increase in organotrophic bacteria abundance and a 71.2% increase in actinobacteria abundance. In the case of the cadmium-contaminated soil, the lower Humus Active dose (CdHA2) caused the count of organotrophic bacteria to increase by merely 2.5% and that of actinobacteria to increase by 38.5% compared to the contaminated but not HA amended soil (Cd). The treatment with the higher tested dose of Humus Active (HA4) applied to the non-contaminated soil resulted in lesser abundance of organotrophic bacteria (a decrease by 24.7% compared to the control soil) and, simultaneously, a significant increase in actinobacteria abundance, i.e., by 124.4% compared to the control soil. The amendment of the cadmium-contaminated soil with the higher HA dose (CdHA4) did not cause any significant change in the count of organotrophic bacteria (a decrease by 3.7%) while increasing the count of actinobacteria by 121.5% compared to the Cd-contaminated soil (Figure 1b).

The analysis of colony development (CD) indices of organotrophic bacteria and actinobacteria grown on solid media (Figure 1c) demonstrated their higher values in the soil amended with the higher tested dose of humic acids (35.1 and 29.6, respectively) and in the control soil (32.5 and 27.1, respectively). Soil treatment with cadmium reduced colony growth dynamics, as indicated by 31.0% and 22.7% lower CD of organotrophic bacteria and actinobacteria, respectively.

In the soil contaminated with cadmium and amended with the lower dose of Humus Active (CdHA2), the CD of organotrophic bacteria decreased by 21.4% compared to the control soil (C), but increased by 13.9% compared to the Cd-contaminated soil not treated with HA.

In the contaminated soil treated with the lower tested dose of HA (CdHA2), the CD of actinobacteria decreased by 15.9% and 35% compared to the Cd-contaminated soil (Cd) and non-contaminated soil (C), respectively. Thus, the lower HA dose did not mitigate the adverse effect of cadmium on the development of actinobacteria colonies. In turn, its higher dose yielded a more positive effect, since the CD value decreased by only 0.8% for organotrophic bacteria and 2.3% for actinobacteria compared to the non-contaminated soil. In the soil contaminated with cadmium and amended with the higher HA rate (CdHA4), the CD value of organotrophic bacteria reached 27.5, i.e., decreased by 15.3% compared to the control soil but increased by 22.8% compared to the Cd treatment. In the case of actinobacteria, CD value reached 26.2 and, thus was lower by 3.5% than in the control soil and higher by 24.9% than in the Cd-contaminated soil.

The ecophysiological diversity (EP) indices of organotrophic bacteria and actinobacteria (Figure 1d) indicate that the first were the least diversified (EP = 0.85) in the CdHA2 treatment and the most diversified in the CdHA4 treatment. Cadmium exerted a significant negative effect on the ecological diversity of Org, whereas the higher HA dose mitigated its adverse effects. In turn, the biodiversity of actinobacteria was negatively affected by both Cd and HA, with their highest EP noted in the control soil (0.94) and the lowest one in the soil from the CdHA4 treatment (0.78).

### 2.2. Effect of Soil Contamination with Cadmium (Cd^2+^) on the Abundance and Diversity of Non-Culturable Bacteria

**Figure 2:**
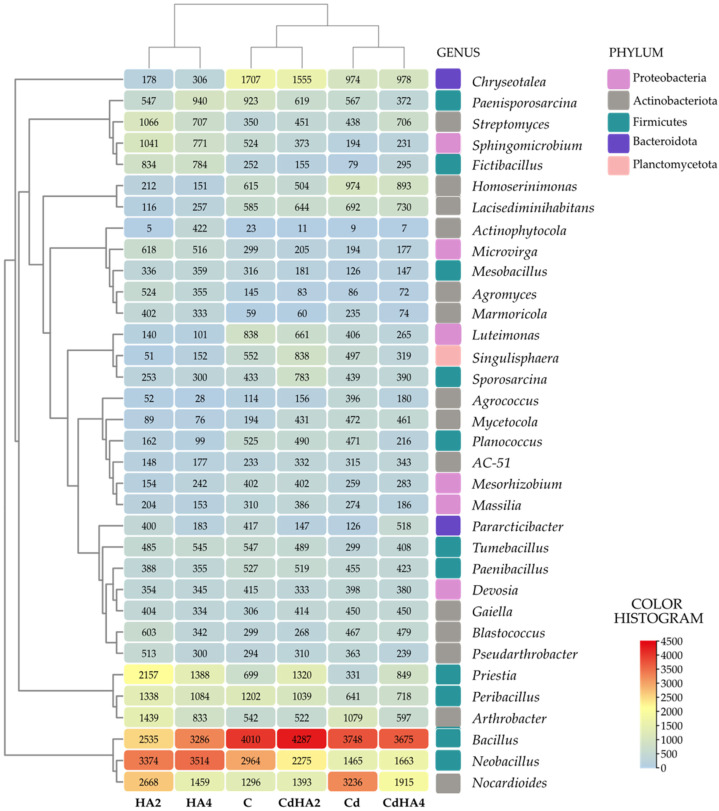
The abundance of bacterial genera in the soil at OTU ≥ 1%. Explanations of the abbreviations used for the treatments are provided in the footnote to Figure 1.

The analysis of the abundance of bacterial genera in the soil (Figure 2) revealed that the prevailing genera included *Bacillus*, *Neobacillus* (classified to the phylum Firmicutes), and *Nocardioides* (classified to Actinobacteriota). Most of the soil samples were predominated by the *Bacillus* genus bacteria, accounting for 20–21% of the entire bacterial population. Similar abundance was noted for the genus *Neobacillus* in the non-contaminated soil amended with the higher Humus Active dose (HA4). The response of these bacteria to soil contamination with cadmium was, however, significantly stronger compared to the *Bacillus* genus bacteria, as their population determined in the soil not amended with HA decreased to barely 8%.

Nonetheless, it is worth emphasizing that Humus Active did promote their abundance (up to 10–11%). The last prevailing genus turned out to be *Nocardioides*, whose population increased substantially after soil treatment with Cd. In turn, soil amendment with the lower Humus Active dose (HA2) increased populations of *Neobacillus* (3374) and *Nocardioides* (2668), while reducing that of *Bacillus* to 2535 sequences. Soil contamination with Cd decreased *Neobacillus* abundance by nearly 50% and that of *Chryseotalea* by 43% compared to the control soil while preserving the high abundance of *Bacillus* (ca. 17%) and nearly doubling the *Nocardioides* population (from 6% to 14%). The amendment of non-contaminated soil with Humus Active (HA2, HA4) significantly modified the bacteriobiome composition. In the HA2 treatment, the contribution of *Neobacillus* in the whole bacteriobiome increased by 14% and that of *Nocardioides* by over 100% compared to the control soil. In the case of the higher HA dose (HA4), the abundance of *Neobacillus* remained the highest among all analyzed soil samples (ca. 16%), and that of *Bacillus* decreased by 18%, compared to the control treatment. The abundance of *Priestia* increased in both treatments with Humus Active, i.e., three times (HA2) and two times (HA4), compared to the control treatment. The soil contaminated with cadmium and amended with the lower Humus Active dose (CdHA2) was predominated by *Bacillus* (ca. 21% of all sequences), whose contribution increased by 14% compared to the Cd treatment. At the same time, analyses showed a 1.5-fold increase in *Neobacillus* and a 1.8-fold in *Priestia* abundance. The bacterial structure of the CdHA4 soil sample was similar, with prevailing *Bacillus* (ca. 19%) and *Nocardioides* (ca. 10%), and with *Neobacillus* abundance reduced to 9%. Results of the new generation sequencing (NGS) analysis additionally draw attention to a diversified structure of the soil bacteriobiome, indicating a significant contribution of typical representatives of the phylum Actinobacteria, including *Streptomyces*, *Arthrobacter*, and *Gaiella*, regardless of experimental treatment. The greatest increase in the contribution of these taxa in the bacteriobiome was noted in the non-contaminated soil treated with the lower Humus Active dose (HA2), with 3-fold and over 2.5-fold increases observed in *Streptomyces* and *Arthrobacter* populations, compared to the control soil.

**Figure 3:**
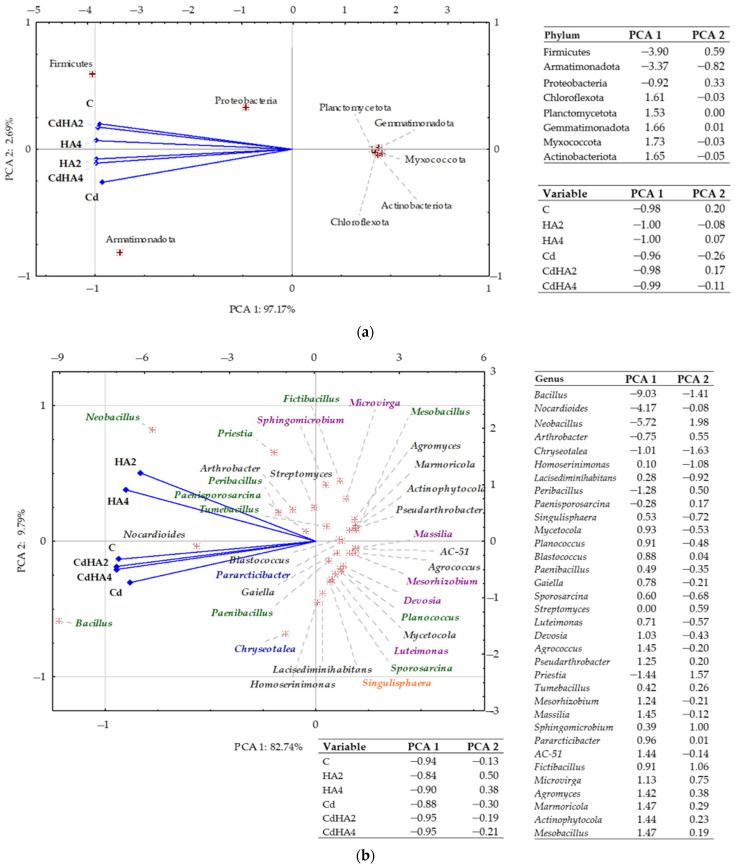
PCA biplot of the diversity of (**a**) bacterial phyla and (**b**) bacterial genera based on OTU data. Explanations of the abbreviations used for the treatments are provided in the footnote for Figure 1.

The principal component analysis (PCA) conducted for bacteria at the phylum level revealed that the first two components (PC1 and PC2) explained ca. 99.86% of the total variance (Figure 3a). The analyzed soils, i.e., C, HA2, HA4, Cd, CdHA2, and CdHA4, grouped in the plot of negative PC1 values (−0.96 to −1.00), which confirms their high homogeneity. In turn, the prevailing bacterial phyla differed significantly. Firmicutes and Armatimonadota were strongly correlated with the soil types (treatments), i.e., C, HA2, HA4, Cd, CdHA2, and CdHA4, and reached high negative PC1 values (−3.90 and −3.37). The third phylum was represented by bacteria belonging to Proteobacteria (−0.92), which suggests their broad ecological spectrum and capability to function in environments not contaminated and contaminated with cadmium. In contrast, the PC1 values of the remaining identified phyla, i.e., Planctomycetota, Gemmatimonadota, Myxococcota, Actinobacteriota, and Chloroflexota, were positive (from 1.53 to 1.73).

In turn, the PCA conducted for bacteria at the genus level demonstrated that the first two principal components explained ca. 90.73% of the total variance (Figure 3b), with PC1 explaining 82.74% and PC2 explaining 7.99%. A clear division of data was also observed along the PCA1 axis. Bacteria from the genus *Bacillus* (−9.03) and *Neobacillus* (−5.72) were strongly correlated with negative PCA1 values, whereas numerous other genera, like *Planococcus*, *Agrococcus*, *Massilia*, *Mesorhizobium*, or *Actinophytocola*, reached high positive PCA1 values (from 0.9 to 1.5).

**Figure 4:**
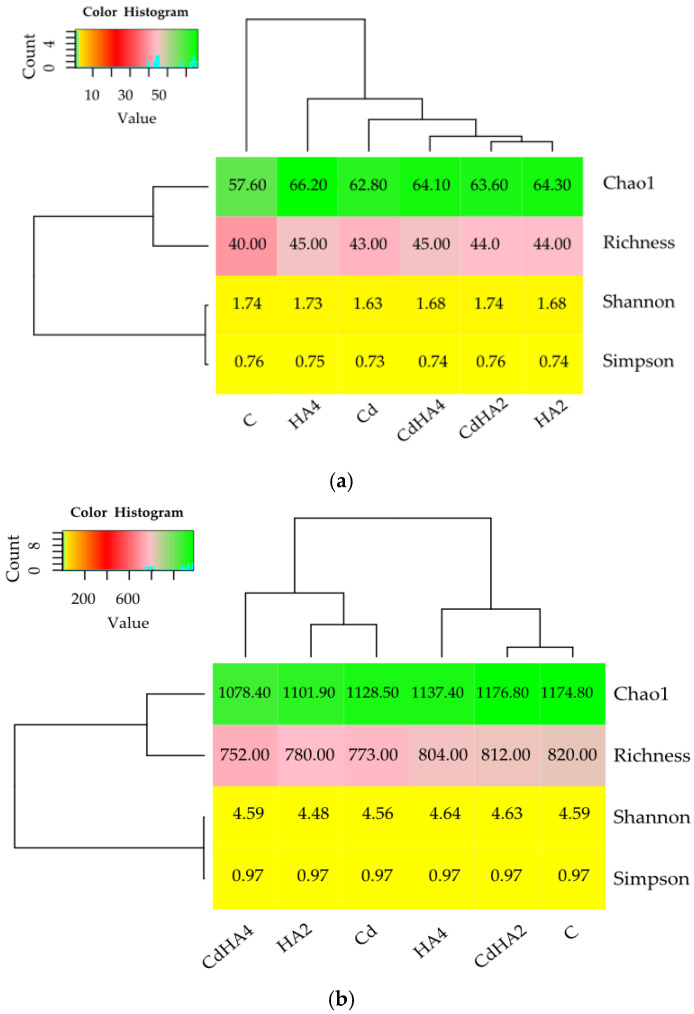
The diversity indices of the bacteria were computed at the phylum (**a**) and genus (**b**) OTU levels. Explanations of the abbreviations used for the treatments are provided in the footnote for Figure 1.

The analysis of the alpha diversity indices demonstrated significant differences between their values at the genus and phylum levels (Figure 4a,b). Values of the Shannon’s diversity index were definitely higher at the genus (4.48–4.64) than at the phylum level (1.63–1.74), pointing to the large diversity of genera and uniform distribution of taxa in the analyzed soil treatments. The highest Shannon’s index at the genus level was noted in the non-contaminated soil amended with HA4 (4.64), and the lowest one in the HA2 treatment (4.48). At the phylum level, its highest value (1.74) was computed in the CD-contaminated soil treated with HA2 (CdHA2) and in the control soil (C), whereas the lowest one was in the Cd-contaminated soil (1.63). These findings confirm Cd’s role as a stressor, reducing both the observed and potential diversity. The Simpson’s index was stable at the genus level (0.97) and did not confirm the predominance of one bacterial genus in the soil microbiome. It was, in turn, less stable at the phylum level, ranging from 0.73 (Cd) to 0.76 (C and CdHA2), which points to a slightly greater predominance of a few main phyla in the control soil and Cd-contaminated soil amended with the lower HA dose (HA2). The species richness at the genus level ranged from 752 (CdHA4) to 820 (C) and was considerably lower at the phylum level, ranging from 40 (C) to 45 (HA4). In turn, values of the Chao1 estimator, taking account of rare taxa, were higher than the observed richness, suggesting the presence of not detected, rare genera called ‘hidden diversity’. The highest Chao1 value was determined at the genus and phylum level in the Cd-contaminated soil amended with the lower HA dose (CdHA2). This finding suggests that the application of the lower HA dose onto the Cd-contaminated soil increases the potential richness of bacteria and contributes to stress mitigation to a greater extent than the higher HA dose. Thus, the higher dose of the HA preparation does not compensate Cd-induced stress as effectively as the lower one does.

### 2.3. Metabolic and Ecological Functions of the Analyzed Bacteria

**Figure 5:**
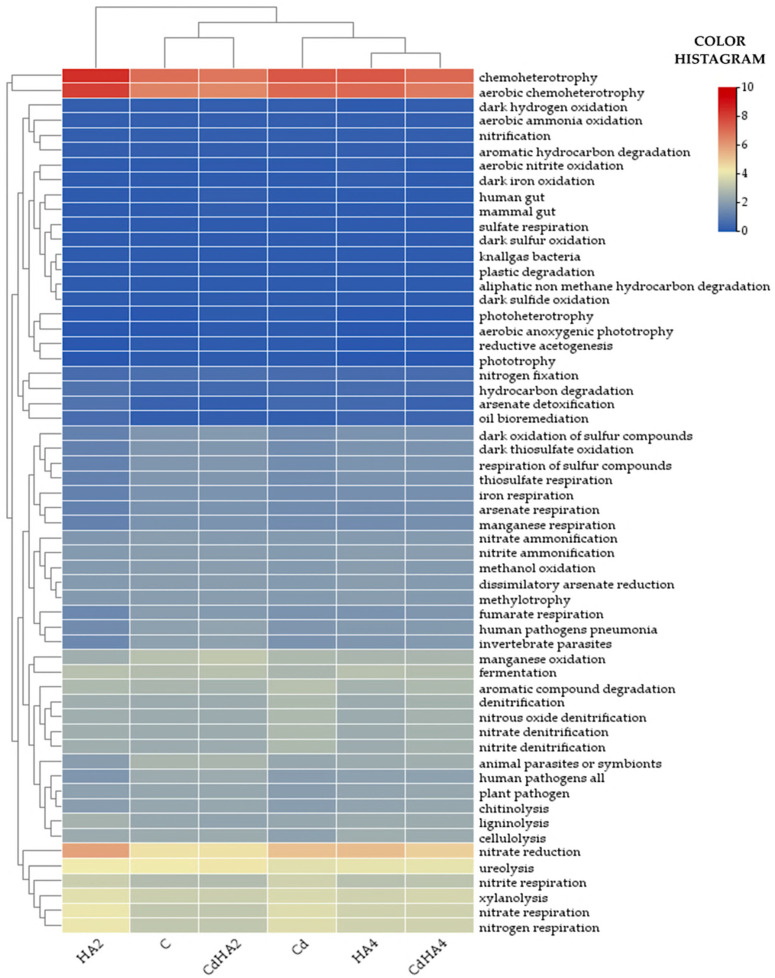
The relative OTU number of bacterial genera with potential metabolic functions. Explanations of the abbreviations used for the treatments are provided in the footnote for Figure 1.

**Figure 6:**
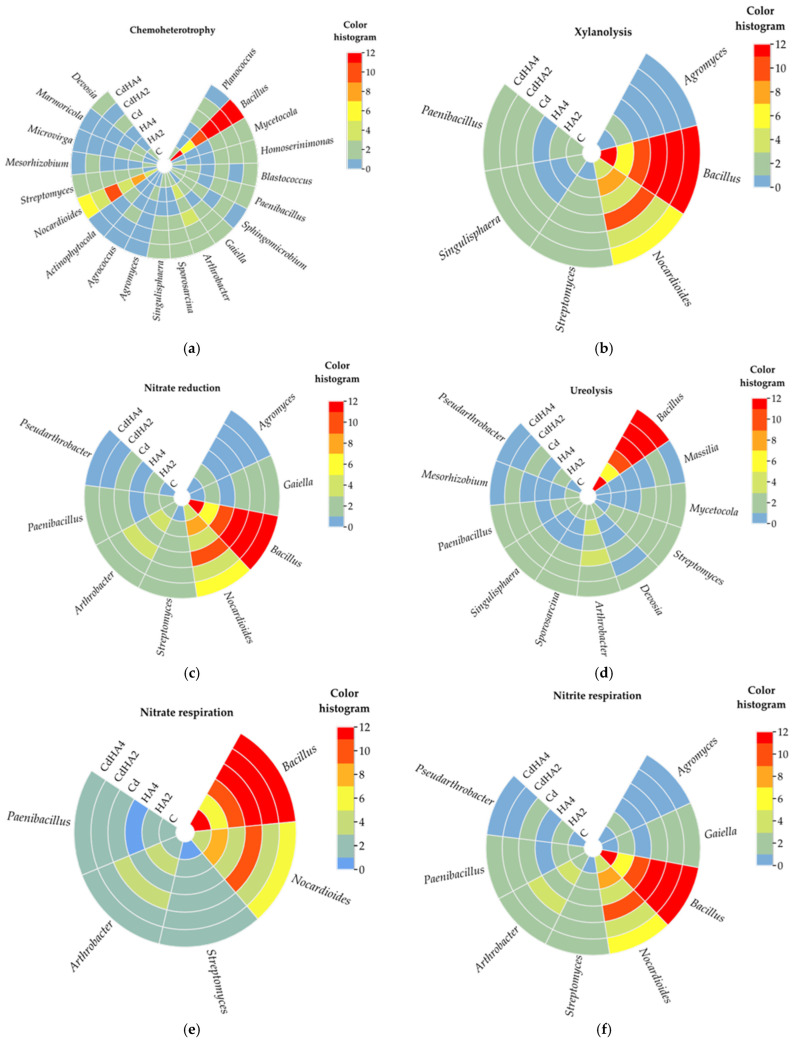
The prevailing genera of bacteria: (**a**) chemoheterotrophic, and involved in (**b**) xylanolysis, (**c**) nitrate reduction, (**d**) ureolysis, (**e**) nitrate respiration, (**f**) nitrite respiration. The color scale denotes the OUT percentage. Explanations of the abbreviations used for the treatments are provided in the footnote to Figure 1.

The analysis conducted using the FAPROTAX software http://www.loucalab.com/archive/FAPROTAX/ (accessed on 16 December 2025) demonstrated that chemoheterotrophs predominated in all analyzed soil samples (Figure 5). Their population ranged from 6.4% in the Cd-contaminated soil treated with the lower dose of the humic acid preparation (CdHA2) to 8.4% in the non-contaminated soil amended with the lower HA dose (HA2) and included bacteria belonging to the following genera: *Planococcus*, *Bacillus*, *Mycetocola*, *Homoserinimonas*, *Blastococcus*, *Paenibacillus*, *Sphingomicrobium*, *Gaiella*, *Arthrobacter*, *Sporosarcina*, *Singulisphaera*, *Agromyces*, *Agrococcus*, *Actinophytocola*, *Nocardioides*, *Streptomyces*, *Mesorhizobium*, *Microvirga*, *Marmoricola*, and *Devosia* (Figure 6a). The key functions of these bacteria sourcing energy from the oxidation of organic compounds and preserving soil fertility also include involvement in relatively intensive processes associated with the breakdown of xylans (xylanolysis), i.e., most hardly degradable biotic polymers. These processes occurred with equal intensity in all analyzed soil samples (from 3.4 to 3.9%) and were mediated mainly by the prevailing bacteria of the *Bacillus*, *Nocardioides*, *Agromyces*, *Singulisphaera*, *Paenibacillus*, and *Streptomyces* genera (Figure 5 and Figure 6b).

Other predominating functions of the analyzed bacterial community included involvement in processes linked with nitrogen cycling (e.g., ureolysis, nitrate reduction, nitrate respiration, nitrogen respiration, nitrite respiration). The nitrate-reducing bacteria exhibited the greatest potential in this respect. Their OTUs accounted for 4.4% (C and CdHA2) to 5.8% (HA2) of all OTUs with the ascribed function. The fraction of OTUs with the potential for ureolysis ranged from 3.9% (Cd) to 4.3% (CdHA2). In turn, the contribution of bacteria involved in nitrate respiration, nitrogen respiration, and nitrite respiration ranged from 2.9% to 4.1% (Figure 5). The least represented functions were those associated with nitrite ammonification and nitrate ammonification (1.8–2%). The predominating bacteria linked with nitrogen cycling were those from the following genera: *Bacillus*, *Nocardioides*, *Arthrobacter*, *Paenibacillus*, *Pseudarthrobacter*, *Gaiella*, *Agromyces*, *Streptomyces* as well as *Massilia*, *Mycetocola*, *Devosia*, *Sporosarcina*, *Singulisphaera*, *Mesorhizobium* (Figure 6c–f).

Other important metabolic functions include the respiration of sulfur compounds, thiosulfate respiration, dark oxidation of sulfur compounds, iron or manganese respiration. However, in the present study, they were ascribed to only 1.2% to 1.9% of the bacteria (Figure 6), as these bacteria were associated with environment detoxication and mobilization of trace elements and included *Alkaliphilus*, *Thermacetogenium*, *Sporanaerobacter*, *Desulfofarcimen*, *Thermosulfidibacter*, *Desulfobacca*, *Desulfosporosinus*, *Garciella*, *Desulfotomaculum*, *Desulfatitalea*, *Desulfohalotomaculum*, *Nitrospira*, *Aciditerrimonas*, *Ardenticatena*, and *Anaeromyxobacter* identified at less than 1% OUT and bacteria from the genus *Bacillus*.

### 2.4. Effect of Soil Contamination with Cadmium on Zea mays Leaf Greenness Index (SPAD)

**Figure 7:**
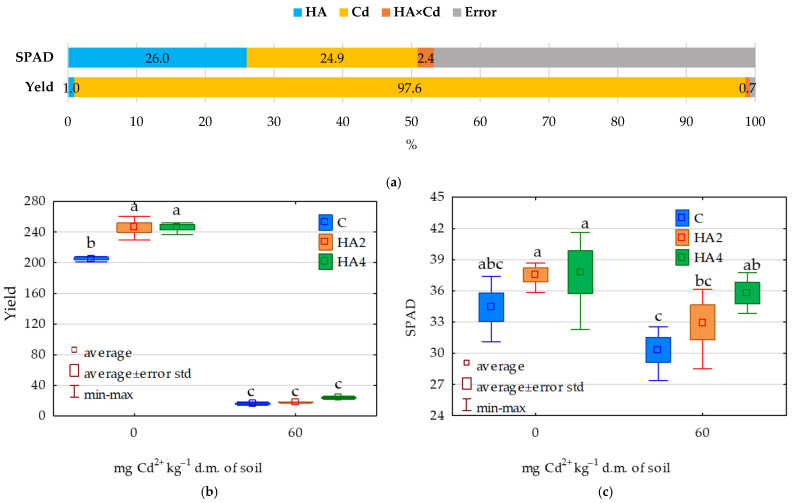
Analysis of the effect size (η^2^) on plant and SPAD (**a**), *Zea mays* (f.m. g per pot) crop yield (**b**) and leaf greenness index (SPAD) of *Zea mays* (**c**). Cd—soil contaminated with cadmium; HA—soil amended with Humus Active preparation. Explanations of the abbreviations used for the treatments are provided in the footnote to Figure 1. a–c—homogeneous groups.

Results of the analysis of variance (Figure 7a) indicate that *Zea mays* biomass was affected to the greatest extent by soil contamination with cadmium, which explained 97.6% of the variability in respective data. The effect of Humus Active application accounted for 1.0% of the total variance, whereas interaction of both experimental factors (HA × Cd) accounted for 0.7%. No prevailing factor was distinguished in the case of the leaf greenness index (SPAD) (Figure 7c), as Humus Active treatment explained 26.0% and soil contamination with cadmium explained 24.9% of variability in SPAD values.

Soil contamination with cadmium (Cd) caused a 92.0% reduction in *Zea mays* biomass and simultaneously decreased the SPAD value by 11.9% compared to the control treatment (Figure 7b). The lower dose of Humus Active (HA2) applied on the non-contaminated soil increased the *maize* biomass by 21.8% and SPAD value by 9.0% compared to the control treatment. In turn, the amendment of Cd-contaminated soil with the lower HA dose (CdHA2) caused a 10.0% increase in *Zea mays* biomass and an 8.7% increase in SPAD value compared to the Cd treatment. In the case of the higher HA dose (HA4) applied on the non-contaminated soil, *maize* biomass increased by 17.2% and SPAD value by 9.8% compared to the control soil. Amendment of Cd-contaminated soil with the higher HA dose (CdHA4) caused a 47.2% increase in *maize* biomass and a 17.9% increase in SPAD value compared to the Cd treatment.

**Figure 8:**
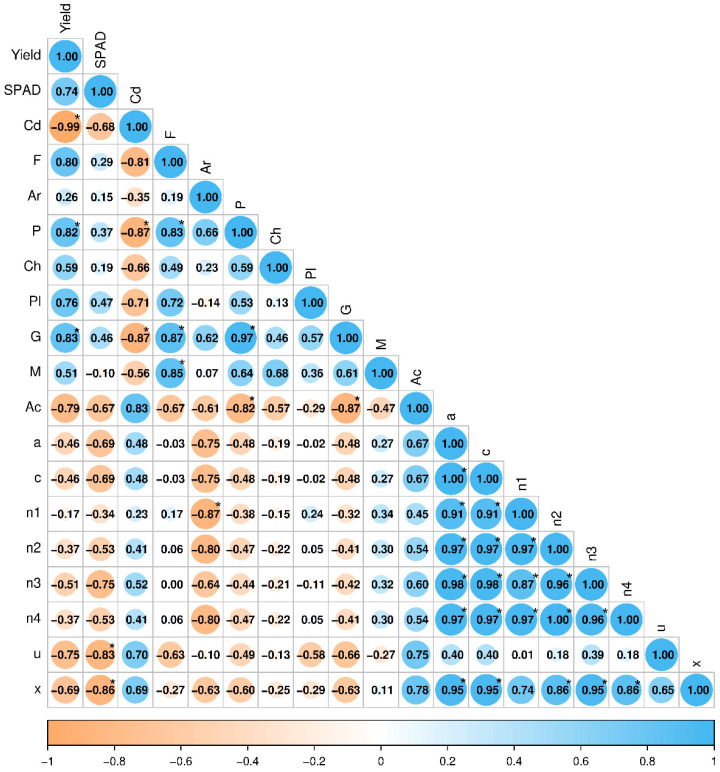
Pearson’s simple correlation coefficients. Significant at *p* = 0.05, n = 9. Red color—statistically significant, black color—statistically insignificant. Explanations of the abbreviations: F—Firmicutes; Ar—Armatimonadota; P—Proteobacteria; Ch—Chloroflexota; Pl—Planctomycetota; G—Gemmatimonadota; M—Myxococcota; Ac—Actinobacteriota; a—aerobic chemoheterotrophy; c—chemoheterotrophy; n1—nitrate reduction; n2—nitrate respiration; n3—nitrite respiration; n4—nitrogen respiration, u—ureolysis; x—xylanolysis, * statistically significant difference (*p* < 0.05).

Correlation coefficients showed that cadmium levels in the soil had a significantly negative effect on *Zea mays* yield (r = −0.99) (Figure 8). There was also a trend towards a negative effect on SPAD (r = −0.68). Cadmium had a negative effect on the growth of bacteria from the phyla Firmicutes (r = −0.81), Proteobacteria (r = −0.87), and Gemmatimonadota (r = −0.87), and a positive effect on the growth of bacteria from the phylum Actinobacteriota (r = 0.83). However, it did not cause any statistically significant changes in bacterial metabolic functions, although the correlation between cadmium and bacterial functionality was consistently positive, ranging from r = 0.23 for nitrate reduction to r = 0.70 for ureolysis.

The yield of *Zea mays* was positively correlated with Proteobacteria (r = 0.82), indicating that bacterial species that enhance the growth of this plant should be sought in this group. Conversely, there was a negative correlation with Gemmatimonadota (r = −0.83). Additionally, positive correlations were observed between Gemmatimonadota and Firmicutes (r = 0.87), as well as between Gemmatimonadota and Proteobacteria (r = 0.97). Conversely, negative correlations were observed between Actinobacteriota and Proteobacteria (r = −0.82), and between Actinobacteriota and Gemmatimonadota (r = −0.87). Significant positive correlations were observed among the bacterial metabolic functions examined, except for ureolysis. Ureolysis and xylanolysis were negatively correlated with the SPAD of *Zea mays* leaves. The respective correlation coefficients were r = −0.83 and r = −0.87.

## 3. Discussion

### 3.1. Effect of Soil Contamination with Cadmium and Amendment with Humus Active on Culturable Bacteria

In our study, both organotrophic bacteria (Org), to a lesser extent, and actinomycetes (Act) were found to be significantly sensitive to cadmium (Cd) stress, which also lends credence and justification to reports by other researchers. According to Syed et al. [43], the reduced microbial abundance in cadmium-contaminated soil results from disruptions in the permeability of microbial cell membranes. Bravo and Braissant [23] also emphasized disturbances in the balance between the generation of harmful free radicals and the organism’s ability to neutralize them. Consequently, damage to proteins, DNA, or lipids could lead to premature aging and cell death. Oxidative stress could also be associated with the specific environmental conditions prevailing in a given habitat. According to Wang et al. [44], changes in electrical conductivity may also affect bacterial growth and reduce their biomass, thereby limiting essential physiological functions such as water uptake and respiration. A significant reduction in microbial abundance in cadmium-amended soils has also been confirmed by You et al. [45] and Deng et al. [46], who demonstrated that cadmium stress led to a decrease in microbial biomass and disruptions in soil activity, consequently promoting the survival of only the most resilient taxa, which may potentially be utilized in bioremediation. Both our study results and previous literature reports [44,47,48,49] indicate that bacterial responses to cadmium contamination proceed dynamically. Therefore, characterizing shifts in the structure of cultivable microorganisms in soil is increasingly being employed as an indicator of soil quality changes [44].

The application of Humus Active to Cd-uncontaminated soil resulted in increased microbial abundance, with a pronounced stimulation of actinobacteria, especially at the lower dose (HA2). This stimulatory effect may be attributed to the supply of organic substrates as well as improvements in soil structure and water-holding capacity [50]. In Cd-contaminated soil, the addition of HA at a dose of 2 g kg^−1^ mitigated the toxicity of this metal to the soil’s microbial properties, exposing the resistance of the actinobacteria. An even stronger effect was observed in the treatment with 4 g HA × kg^−1^ (CdHA4), where the abundance of Act more than doubled (121%) compared to the Cd-contaminated soil without HA amendment. Such a positive response of actinobacteria to a compilation of soil contamination with cadmium and humic acids is due to several proven facts based on the synergistic action of several adaptive mechanisms. The advantage over organotrophic bacteria is given by the ability proven in Actinobacteria to duplicate metal resistance genes, including the copD gene [51]. In addition, Actinobacteria efficiently metabolize complex organic compounds such as humates and fulvacids, giving them a competitive advantage over organotrophic bacteria, which prefer simple carbon sources. In a study by Bao et al. [52], about 16% of genes assigned to Actinobacteria encoding carbohydrate-degrading enzymes (CAZymes) were responsible for this potential. These findings are consistent with the results reported by Kou et al. [53], Zaborowska et al. [54], and Zhou et al. [55], who showed that humic compounds reduced cadmium bioavailability through complexation of Cd^2+^ ions. Furthermore, soil amendment with humic acids promotes microbial diversity and stimulates microbial groups capable of metal detoxification. In the present study, the supplementation of cadmium-contaminated soil with Humus Active contributed to the partial restoration of organotrophic bacteria and actinobacteria abundance, which is consistent with the findings of Aguilar-Paredes et al. [56] and Wyszkowska et al. [57].

Analysis of functional properties of cultivable microorganisms revealed that their colony development (CD) and ecophysiological diversity (EP) indices were markedly reduced in Cd-contaminated soils while remaining stable in the other treatments. This finding supports the hypothesis that stress conditions suppress microbial activity [58,59].

The application of Humus Active on the non-contaminated soil increased CD values in the studied bacterial groups, indicating their improved capacity for rapid colonization. These observations align with previous studies [58,60,61], which demonstrated that improving soil conditions in contaminated systems might stimulate microbial development; however, full restoration of functional diversity required removal or substantial reduction in the stress factor. Maji et al. [62] and Li et al. [63] mention that the phenomenon of stimulation may correspond to the formation of microenvironments and the provision of soluble carbon sources. In the present study, the application of Humus Active to the Cd-contaminated soil increased the CD index from 22.43 to 25.55 (CdHA2) and 27.54 (CdHA4), confirming that this amendment partially restored bacterial diversity in the cadmium-contaminated soils. Wei et al. [64] reached similar conclusions for heavy metal-contaminated soils, where humic substances increased the abundance of metal-resistant and detoxifying bacterial taxa.

### 3.2. Effect of Soil Contamination with Cadmium and Amendment with Humus Active on the Abundance of Non-Culturable Bacteria

A more detailed metagenomic analysis of the soil bacterial community showed *Bacillus*, *Neobacillus*, and *Nocardioides* to be the dominant genera. This finding is consistent with earlier reports by Cheng et al. [65] or Song et al. [66], indicating that members of the Firmicutes and Actinobacteriota phyla exhibit high tolerance to heavy metals, including cadmium. In the present study, cadmium-induced stress reduced the abundance of the dominant phyla Firmicutes, Proteobacteria, and Bacteroidota while increasing the population of Actinobacteriota. Sun et al. [67], Cheng et al. [65], and Wang et al. [68] also suggested that even low Cd concentrations (0.5–2 mg × kg^−1^) could reduce the abundance of sensitive taxa, particularly those belonging to Proteobacteria, Bacteroidota, and Firmicutes phyla while increasing the proportion of Actinobacteriota, which exhibit greater resistance to metal-induced stress [66]. However, this pattern is not consistently observed, as Lin et al. [69] reported a significant decrease in the abundance of Actinobacteriota (by 13.4%) and Firmicutes (by 12.8%) at increasing cadmium concentrations in soil. Among these, *Bacillus* strains were identified through functional prediction analysis as key biomarkers in monitoring cadmium contamination. Similarly, in the present study, *Bacillus* was the dominant genus under stress conditions. Its high abundance, accounting for 21% of all detected sequences, could be associated with its capability for active chemisorption and bioaccumulation of Cd ions through P-type ATPase pumps and RND protein transporters. A similar mechanism has been indicated by Sharma et al. [33], Adhikary et al. [70], and Rolón-Cárdenas and Hernández-Morales [71]. These mechanisms enable cells to remove excess metal cations from the cytoplasm, thereby mitigating their toxic effects. Additionally, these bacteria often synthesize metallothioneins and exopolysaccharides (EPS), which form a protective barrier on the cell surface, impeding the penetration of metal ions [23]. The observed increase in the relative abundance of *Nocardioides*, belonging to the phylum Actinobacteriota, in cadmium-contaminated soils may indicate their active involvement in cadmium bioconversion [23]. Zhang et al. [72] demonstrated that bacteria from this group also secrete extracellular polymeric substances (EPSs) and siderophores, enhancing the immobilization of heavy metals. They may be involved in bioweathering and bioleaching, converting Cd^2+^ into less mobile forms such as CdS, CdCO_3_, or Cd(OH)_2_ [72,73]. This mechanism leads to metal immobilization and its reduced bioavailability to plants, which is of fundamental importance for the protection of agroecosystems.

By increasing the sorption capacity of soil and availability of carbon sources, humic acid can enhance the activity of microorganisms involved in bioleaching and biosorption [74,75]. The increased abundance of bacteria belonging to *Neobacillus* and *Nocardioides* genera demonstrated in the present study after soil amendment with the Humus Active preparation (HA2) may be indicative of the synergistic action of organic compounds and soil microbiota during Cd removal from the environment. Furthermore, increased counts of *Streptomyces*, *Arthrobacter*, and *Gaiella* in the soil treated with the lower Humus Active dose (HA2) point to the activation of functional groups associated with carbon and nitrogen cycling and to increased potential for Cd bioconversion through the synthesis of chelating agents (EPS) [70,71,76]. Actinobacteria, known for their capability to biosynthesize complex secondary metabolites, can aid biostimulation or bioweathering and the formation of stable Cd complexes in soil [77,78].

HA interaction with bacteria may trigger shifts in the expression of genes associated with mechanisms of resistance to metals—i.a. genes encoding efflux systems and pathways responsible for biosynthesis of EPS and metalloproteins (metallothioneins) [79,80,81]. Such changes are due to both modified Cd availability (lesser stress of free ions) and the supply of additional sources of carbon/metabolic stimulants by HA, which promotes intensifications of EPS synthesis and biofilm formation—being of key importance to bioleaching and biosorption. However, the ultimate effect depends on preparation dose and quality, i.e., moderate HA concentrations often enhance microbiological diversity and remediating functions, whereas excess HA rates may shift bacterial community composition towards groups such as Actinobacteria, thereby exerting a negative effect on certain functions, like N and P biogeochemical cycling [82,83]. Furthermore, humic acids represent a source of carbon easily available to heterotrophic bacteria. They improve water retention and cationic exchange, thus promoting bacteria development. In lower doses, they stimulate microbiological diversity and support populations involved in N biogeochemical cycling, whereas their overly high doses may induce short-term suppression of the redox activity of microorganisms due to excess complexation of trace elements [74,76,84]. Appropriately selected HA doses exert a biostimulating effect by activating genes responsible for carbon metabolism and EPS synthesis, but do not induce mechanisms of resistance to metals.

Hence, under conditions of soil contamination with cadmium, humic acids play a dual—chemical and biological—role. On the one hand, their molecules, rich in carboxyl and phenolic groups, complex Cd^2+^ ions, reducing their bioavailability and toxicity, and support the formation of biofilms and sorptive microstructures that bind Cd into less mobile forms. In this way, humic acids not only mitigate metal-induced stress but also shift the dominant adaptive strategies of bacteria from the defensive (intracellular detoxification) to the environment-stabilizing ones (biosorption, biomineralization). The synergistic interaction between humic acids and Cd-tolerant microorganisms currently represents one of the most promising approaches in the biological remediation of soils contaminated with heavy metals [67]. Results of the present study clearly demonstrate that soil contamination with Cd significantly modifies soil bacterial community structure, whereas the application of a humic preparation promotes the recovery of bacterial populations with bioremediating potential. The persistence of genera such as *Bacillus*, *Neobacillus*, and *Nocardioides* determines their key role in natural detoxification processes within the soil environment, particularly through bioleaching, biosorption, and biomineralization mechanisms. The use of these microorganisms in combination with natural organic substances may therefore offer an effective and environmentally sound strategy for reducing cadmium mobility in agroecosystems.

### 3.3. Effect of Cadmium and Humus Active on the Soil Microbiome, Bacterial Metabolic Functions, and the Biomass of Zea mays as Well as Leaf Greenness Index (SPAD)

The excessive presence of cadmium in the soil has led to a decrease in bacterial diversity, which is also consistent with the research results of other researchers [26,67,85]. In the present study, the addition of humic acids (HAs) partially mitigated the adverse effects of Cd, increasing both the Shannon index and the Chao1 estimator. The high Chao1 values compared to observed richness—particularly at the genus level—indicate the presence of numerous rare taxa that may perform key ecological functions in soil, such as participating in metal detoxification or the degradation of complex organic compounds [45,86,87,88,89]. Investigations conducted by Ma et al. [90], Xiong et al. [91], and Xiao et al. [92] have shown that unique taxa constitute an essential component of ecosystem stability and its adaptive capacity under environmental stress conditions. Our study findings demonstrate that Cd acts as a stressor reducing bacteriobiome diversity, whereas humic acids may function as natural biostimulants, supporting the restoration of bacterial diversity and enhancing potential species richness. The high Chao1 value underscores the ecological importance of unique taxa in preserving ecosystem functions, which should be taken into account when developing remediation strategies for soils contaminated with heavy metals.

According to Sansupa et al. [93] and Yang et al. [94] the FAPROTAX database can serve as an effective and rapid tool for the tentative ‘functional screening’ of soil-derived 16S rRNA datasets, indicating the functions of bacteria in the soils we studied. This approach seems justified, as bacteria regulate a broad range of soil ecological functions and are essential for assessing the biogeochemical cycling of carbon, nitrogen, and sulfur [90,94]. In our study, all soil samples were dominated by chemoheterotrophs, associated with carbon and nitrogen metabolism, which is probably due to the fact that microorganisms with a wide range of metabolic pathways are able to survive environmental stress, as are specialized taxa [95], Wang et al. [96], Wang et al. [97], and Sansupa et al. [93]. In turn, Wang et al. [98] demonstrated significant correlations between bacteria belonging to *Thermodesulfobacteriota*, *Nitrospirota*, *Patescibacteria*, and *Verrucomicrobiota* genera, which are actively involved in carbon metabolism. However, it should be added that the structural and functional changes of bacteria can also be significantly determined by environmental conditions, including fertilization [72,97].

In parallel, we observed that Cd strongly reduced the growth of *Zea mays*, causing a decrease in biomass and lower SPAD index, which was probably due to interference with chlorophyll synthesis and the uptake of key micronutrients such as Mg^2+^, Fe, and Mn [99,100,101,102,103,104,105]. Lower chlorophyll content leads to reduced photosynthetic efficiency and oxidative stress, which directly translates into reduced *Zea mays* biomass production [102,103]. There is also no doubt that cadmium is defined as one of the most toxic heavy metals for plants, and the mechanism of toxicity of this heavy metal is complex and multidirectional [99,101]. The application of Humus Active resulted in an improvement in the plant’s condition, determined by an increase in its biomass in a dose-dependent manner. The higher dose clearly increased this parameter and SPAD values, while the lower dose generated a significant but moderate improvement. Based on scientific reports, it can be assumed that the mechanism of this action was twofold: reduction in Cd bioavailability due to the formation of complexes with metal ions, and the biostimulation of growth through the improvement of soil structure, development of the root system, and enhancement of microbial activity [37,106,107,108,109].

It is worth mentioning that relevant to our research, there is also a relationship between microbiome activity in soil subjected to cadmium pressure and soil biostimulation with humic acids and plant biomass. Our research shows that representatives of Actinobacteria are distinguished by their bioremediation potential. It can be assumed that they also positively influenced the growth of *Zea mays* biomass, which was authenticated by the reports of Zhang et al. [110]. The researchers showed that the *Streptomyces rochei* L1 strain is capable of breaking down high-molecular-weight humic acids into smaller aliphatic and aromatic compounds. This process not only modifies the soil environment and increases the availability of organic compounds, but it also stimulates the growth and yield of *Zea mays*, indicating a synergistic effect of HAs-metabolizing microorganisms on soil function and plant health. The genus Bacillus, which was identified in our experiment, probably also exerted an important influence on this correlation. This is evidenced by the results of Shahzad et al. [111], who, by inoculating *maize* seeds with *Bacillus pumilus* in cadmium-contaminated soil, achieved a significant improvement in growth germination and plant biomass, and, more importantly, a reduction in the accumulation of this metal in the roots and shoots.

It is also noteworthy that the ability of humic acids to immobilize heavy metals corresponds to the fact that they have numerous functional groups (e.g., carboxylic, phenolic) with high affinity for metal cations. They thus form stable, insoluble or poorly soluble complexes and chelates with Cd^2+^ ions in soil solution [106,107]. Consequently, this leads to a reduction in the bioavailability of cadmium to plants, limiting its uptake by the roots and transport to aboveground parts. The significantly higher efficacy of the HA4 dose suggests that under conditions of severe contamination, the sorption capacity of the lower dose (HA2) was insufficient to bind the toxic metal pool to a degree that would significantly improve plant condition.

Importantly, the Humus Active formulation also showed a biostimulatory effect in unpolluted soil, increasing both the biomass of *Zea mays* and the value of the SPAD index. These indications underscore the second, remediation-independent mechanism of action of humic substances. This is because they can act similarly to auxins, stimulating root system development, which translates into more efficient water and nutrient uptake [109,112,113,114,115]. The increase in SPAD values recorded in the HA2 and HA4 treatments suggests an improvement in the plant nitrogen nutritional status, which is directly correlated with the chlorophyll content and photosynthetic potential [116,117]. Overall, the results of Humus Active application indicate its dual, positive mechanism of action: as a growth biostimulant under optimal conditions and as an effective remediating agent, mitigating stress induced by cadmium toxicity through its immobilization in the soil.

Observed changes in microbiome abundance and function, as well as plant responses, highlight the complex interactions between the chemical properties of heavy metals, the availability of organic matter and the adaptive strategies of microorganisms. They point to the coexistence of protective, adaptive and remedial processes that shape the stability and functionality of soil ecosystems. Accordingly, our results confirm the potential of Humus Active (HA) as an effective and ecological tool for detoxifying Cd^2+^-contaminated soils. Humus Active acts as a powerful biostimulator for actinomycetes and bacteria, which are crucial in nitrogen cycling and organic matter degradation, processes that directly affect the structure of soils their quality and availability of nutrients for plants.

## 4. Materials and Methods

### 4.1. Characteristics of the Soil Material

Soil samples were collected for analyses from an agricultural area of the Olsztyn Lake District (53.710° N, 20.4320° E), from the top soil layer of 0–20 cm. The fraction composition of the soil was that of loamy sand and included (in %): sand—73.46, silt—24.29, and clay—2.25. The soil was classified as Eutric Cambisol [118], and had the following characteristics (per 1 kg): organic carbon content (C_org_)—6.95 g, total nitrogen content (N_Total_)—1.06 g, Cd content—0.75 mg, hydrolytic acidity (HAC)—5.5 mmol^(+)^, sum of exchangeable base cations (EBC)—140.0 mmol^(+)^, sorption capacity (CEC)—145.5 mmol^(+)^, saturation with base cations (BS)—96.2%, and pH_KCl_ 6.3.

### 4.2. Cadmium

Cadmium(II) sulfate, with the formula 3CdSO_4_ × 8H_2_O, was purchased from an American company (Sigma-Aldrich, Saint Louis, MO, USA) owned by the Merck Group (Merck KGaA, Darmstadt, Germany). It is characterized by good stability at room temperature and good solubility in water, and is therefore frequently used in environmental and toxicological studies. Upon dissolution, it dissociates into Cd^2+^ cations and SO_4_^2−^ anions. It is a highly toxic and carcinogenic compound that accumulates in the environment and living organisms, causing metabolic disorders. In soil conditions, cadmium sulfate introduced in this form occurs primarily in the ionic form, which facilitates its mobility and bioavailability for microorganisms and plants. Therefore, it is a suitable model compound for assessing the effect of Cd^2+^ ions on microbiological processes in soil.

### 4.3. Characteristics of Humus Active Soil Biostimulant

Humus Active “Humic Acids”, a pulp for allotment gardens, contained approximately 55% humic acids, including 49% of humic fraction, 1% of fulvic acids and 5% of humins and ulmins. Its composition is as follows: nitrogen (N) 0.02% (m/m); phosphorus (P_2_O_5_) 0.30% (m/m); potassium (K_2_O) 0.55% (m/m); magnesium (MgO) 0.05% (m/m); calcium (CaO) 0.30% (m/m); iron (Fe) 450 mg × kg^−1^; manganese (Mn) 15 mg × kg^−1^; zinc (Zn) 3 mg × kg^−1^; and copper (Cu) 1 mg × kg^−1^. According to the producer’s declaration (EKODARPOL, Poznań, Poland, https://www.ekodarpol.pl/ (accessed on 16 December 2025), this stimulant also contains proteins, amino acids, and colloidal silica.

### 4.4. Experiment Design

**Figure 9:**
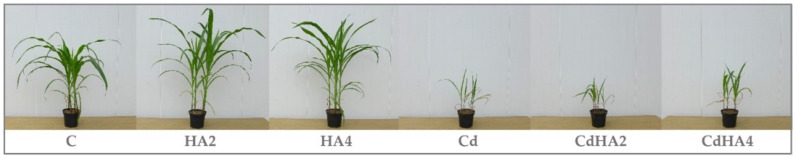
*Zea mays* growth at day 60.

The research experiment was carried out in the split plot design, in 4 dm^3^ polyethylene pots, in four replicates. Two experimental factors were investigated: (1) cadmium dose (cadmium(II) sulfate, 3CdSO_4_ × 8H_2_O: 0 and 60 mg Cd^2+^ per 1 kg of soil), and (2) soil stimulant dose (Humus Active (HA) doses of 0, 2, and 4 g per 1 kg of soil). Basic fertilization with N, P, K, and Mg in the form of aqueous solutions of CO(NH_2_)_2_, KH_2_PO_4_, KCl, MgSO_4_ × 7H_2_O with soil (3.4 kg) was applied in all experimental treatments (non-contaminated soil, Cd-contaminated soil, without and with HA amendment). The fertilization rates were the same in all treatments and reached (in mg per 1 kg d.m. of soil): N—150, P—50, K—150, and Mg—20. The fertilization was followed by soil amendment with Cd^2+^ and HA in respective treatments. *Maize* (*Zea mays*) of DS1897B cultivar served as the test plant (Figure 9) and was grown in each pot (4 plants per pot) throughout the study period, i.e., 60 days. The soil was hydrated with demineralized water to 60% moisture content. Plants were harvested at the BBCH59 stage (Biologische Bundesanstalt, Bundessortenamt and Chemical).

### 4.5. Methodology of Chemical and Physicochemical Analyses of Soil

Soil properties, including organic carbon content (C_org_) and total nitrogen content (N_Tot_), were determined with a TOC-5000 analyzer (Shimadzu, Kyoto, Japan); whereas soil pH in 1 mol KCl dm^−3^ was measured using an HI 2221 pH-meter (Hanna Instruments, Washington, UK) (soil pH) [119]. Hydrolytic acidity (HAC) and the sum of exchangeable based cations (EBC) was determined with the Kappen methods [120,121] described in our previous works [59,122,123]. Data obtained were used to compute sorption capacity of the soil (CEC) and its saturation with base cations (BS) using the following formulas: CEC = HAC + EBC; and BS = EBC/CEC × 100. Cadmium content of the soil was determined according to the Polish Standard PN-ISO 11466:2002 [124]. All determinations were conducted in four replicates.

### 4.6. Methodology of Microbiological Analyses of Soil

Culturable bacteria, i.e., organotrophic bacteria (Org) and actinobacteria (Act), were determined with the method of serial dilutions using the Bunt and Roviry culture medium [125] for Org, and Küster and Williams culture medium [126] for Act. Soil samples were suspended in a physical saline solution (0.85% NaCl, 1:10 (*m*/*v*)), and bacteria were isolated with the deep inoculation method (four replicates). Bacterial cultures were incubated at a temperature of 28 °C for 10 days (Selecta Incudigit, Barcelona, Spain). Results were expressed as cfu × kg^−1^ d.m. of soil. The determined counts of culturable bacteria enabled computing their colony development (CD) index and ecophysiological diversity (EP) index [127,128].

Non-culturable bacteria were identified based on the V3–V8 region of the 16S rRNA gene. Amplification of a selected region of a bacterial population was performed using universal primers 337F and 1391R. The resulting amplicons were sequenced in the nanopore technology using the native barcoding 1D protocol, whereas taxonomic classification was conducted by means of Ublast algorithms, QIIME2 platform, and the GreenGenes2 reference database v2024.1 [129]. The new generation sequencing (NGS) procedure was performed by the genXone company (genXone, Złotniki, Poland).

### 4.7. Data Analysis and Statistical Analysis

Cadmium toxicity against *Zea mays* was analyzed based on the biomass size and leaf greenness index (SPAD), determined using a 502 Chlorophyll Meter (model 2900P, Spectrum Technologies, Thayer, Aurora, IL, US). The results were subjected to statistical analysis using Statistica ver. 13.3 software [42]. HSD Tukey’s test was applied to distinguish homogenous groups (at a significance level of *p* ≤ 0.05.). The percentage contribution of variables associated with particular experimental factors was determined for both the bacteria and plants by means of a two-way analysis of variance (ANOVA) using the eta^2^ coefficient.

The effect of cadmium on culturable and non-culturable bacteria was assessed based on the colony forming units (cfus) and the number of taxonomic units (OTUs). The cfu data were subsequently used to compute bacterial colony growth and development (CD) indices and their ecophysiological diversity (EP). Data were imaged using TBtools-II v2.376 [130].

Metagenomic data served to calculate the Shannon’s diversity index, Simpson’s index, Richness index, and Chao1 estimator [92,131,132,133,134]. All data achieved for non-culturable bacteria were imaged and statistically processed using R v1.2.5033 software (Boston, MA, USA) with R v3.6.2 [135,136]. In order to present the predominant bacteriobiome and metabolic functions of the analyzed bacteria, readings showing OTU < 1% were eliminated from metagenomic data. Metabolic functions of the bacteria were verified with Macadam software [137,138], whereas their functional roles were determined using the following databases: Functional Annotation of Procaryotic Taxa, FAPROTAX [93,94,139], and IJSEM database of phenotypic data [140]. Pearson r correlation coefficients (*p* < 0.05) were presented using SRplot http://www.bioinformatics.com.cn/ accessed on 16 December 2025 [141].

## 5. Conclusions

Cadmium represents one of the major threats to the quality of agricultural soils. Cd-tolerant bacteria play a key role in biogeochemical processes and may be employed in the bioremediation of contaminated ecosystems. At the same time, the application of humic substances, including the commercial preparation Humus Active (HA), constitutes a promising approach for reducing cadmium bioavailability while improving soil biological properties.

The study demonstrates that cadmium exerted a strong adverse impact on soil bacteria, drastically reducing the abundance of organotrophic bacteria and inhibiting the growth of sensitive species within the genus *Neobacillus*. Simultaneously, these stress conditions selectively favored bacteria belonging to the genus *Nocardioides*. The Humus Active preparation used in the experiment acted as a strong biostimulant of culturable actinobacteria as well as representatives of *Bacillus*, *Priestia*, *Streptomyces*, and *Arthrobacter* genera. The dominant functional genes were primarily associated with chemoheterotrophic bacteria. The identified microorganisms were mainly involved in nitrogen cycling and organic matter degradation.

From the phytoremediation perspective, the higher tested dose of HA (HA4) was more effective than its lower dose (HA2), increasing *Zea mays* biomass by 47.2% compared with Cd-contaminated soil without amendment. Overall, HA was found not to restore the original structure of the soil bacteriobiome but instead, actively restructured it, promoting the development of a new community capable of adapting to stress conditions. Genera such as *Nocardioides*, *Streptomyces*, *Arthrobacter*, and *Bacillus* exhibit high application potential and may serve to formulate microbial consortia for bioremediation strategies. This aligns with research priorities emphasized by FAO, UNEP, and Horizon Europe within the ‘Soil Health and Food’ thematic area.

It should also be emphasized that a limitation of the present study was the use of simulated gradients of cadmium contamination, which, despite ensuring control of the experimental conditions, do not fully capture the complexity of processes occurring in naturally contaminated soils, and future research should focus on an integrated approach combining chemical and microbiological analyses, including detailed determination of cadmium mobility and speciation under varying conditions. It is also important to identify the genes and metabolic pathways of microorganisms and plants involved in its accumulation and detoxification.

## Data Availability

The original contributions presented in this study are included in the article. Further inquiries can be directed to the corresponding author.

## Abbreviations

The following abbreviations are used in this manuscript: Org—organotrophic bacteria; Act—actinobacteria, C—non-contaminated soil; Cd—soil contaminated with cadmium; HA2—Humus Active dose of 2 g per kg of soil; HA4—Humus Active dose of 4 g per kg of soil; CdHA2—soil contaminated with cadmium and amended with a Humus Active dose of 2 g per kg of soil; and CdHA4—soil contaminated with cadmium and amended with a Humus Active dose of 4 g per kg of soil; F—Firmicutes; Ar—Armatimonadota; P—Proteobacteria; Ch—Chloroflexota; Pl—Planctomycetota; G—Gemmatimonadota; M—Myxococcota; Ac—Actinobacteriota; a—aerobic chemoheterotrophy; c—chemoheterotrophy; n1—nitrate reduction; n2—nitrate respiration; n3—nitrite respiration; n4—nitrogen respiration, u—ureolysis; x—xylanolysis.
